# Fading Away: A Case Report on Whipple's Disease

**DOI:** 10.7759/cureus.78423

**Published:** 2025-02-03

**Authors:** Nuno Carvalho, Sofia Miguelote, José Pimenta, Isabel Trindade, Jorge Cotter

**Affiliations:** 1 Internal Medicine, Hospital da Senhora da Oliveira, Guimarães, PRT; 2 School of Medicine, University of Minho, Braga, PRT; 3 Internal Medicine, Hospital Cuf Porto, Porto, PRT; 4 Physical Medicine and Rehabilitation, Hospital da Senhora da Oliveira, Guimarães, PRT

**Keywords:** arthralgia, bacteria, diarrhoea, infectious diseases, macrophages, tropheryma whipplei, weight loss, whipple disease

## Abstract

Whipple’s disease (WD) is a rare, multisystemic chronic disease caused by Gram-positive bacteria *Tropheryma (T.) whipplei*. Transmission usually occurs by the fecal-oral route, as the bacillus has been identified in stagnant water, human feces, and soil. This disorder not only causes malabsorption in the gastrointestinal tract but also has cardiovascular, neurological, ophthalmic, and musculoskeletal effects. Prolonged symptoms are the hallmark of this pathology. Definitive diagnosis requires histologic demonstration of Periodic Acid-Schiff (PAS) staining in macrophages from small bowel mucosa and/or polymerase chain reaction identification of *T. whipplei. *Treatment requires prolonged antibiotics for up to two years.

We present the case of a 69-year-old man, admitted to the emergency department with complaints of worsening nocturnal diarrhea within the last seven months. Fatigue, weight loss, night sweats, and worsening lower limb edema were also present within the same timeframe. On physical examination, palpable lymph nodes were noted. Blood tests displayed hypochromic/microcytic anemia, hypoalbuminemia, and mildly elevated C-reactive protein. A thoracoabdominal computed tomography scan unveiled bilateral pleural effusion, as well as multiple abdominal lymphadenopathy. Upper digestive endoscopy revealed marked duodenal mucosa edema, congestion, and diffuse punctiform lymphangiectasia. Hematoxylin-eosin (HE) and PAS histologic staining of biopsied samples revealed spongy macrophages in lamina propria suggestive of WD. Antibiotic therapy was started with intravenous ceftriaxone for two weeks, followed by sulfamethoxazole/trimethoprim up to one year. After 12 months of antibiotic therapy, clinical improvement was remarkable and the patient fully recovered.

Whipple’s disease is a rare condition and its diagnosis is challenging. Prompt symptomatic recognition and diagnostic workup could avoid delaying appropriate therapy, therefore leading to better outcomes.

## Introduction

Whipple’s disease (WD) is a rare multisystemic chronic disease caused by Gram-positive bacteria *Tropheryma (T.) whipplei*, family *Actinobacteria*. Beyond gastrointestinal malabsorption, this disorder also has cardiovascular, neurological, ophthalmic, and musculoskeletal manifestations [1].

It was initially described by George Whipple in 1907 while reporting the case of a 36-year-old man presenting with diarrhea, weight loss, fever, and migratory polyarthritis. Further documentation of mesenteric lymphadenopathy and skin pigmentation led to its initial designation as “intestinal lipodystrophy” [1,2]. Only 40 years after bacteria identification inside macrophages in submucous tissue was the first patient successfully treated using antibiotics [2].

## Case presentation

We present the case of a 69-year-old man who resorted to the emergency department (ED) with complaints of gradually worsening diarrhea for the last 7 months. Four years prior to the current admission, intermittent dorsolumbar inflammatory arthralgia, with scarce response to non-steroidal anti-inflammatory medication, was referred. Then, seven months before ED admission, the patient presented worsening nocturnal watery diarrhea (10 to 15 stools daily) without mucus, blood, or abdominal pain, along with fatigue, significant weight loss (approximately 30 kilograms), night sweats, and worsening lower limb edema. There was no dyspnea, nausea, vomiting, anorexia, or fever.

On physical examination, the patient had an emaciated appearance, discolored mucous membranes, skin hyperpigmentation, and symmetrical edema up to two-thirds of both legs. Non-adherent, elastic, and painless inguinal lymph nodes, two centimeters in diameter, were palpable. Blood tests revealed severe hypochromic/microcytic anemia, along with leucopenia, moderate hypoalbuminemia, and mildly elevated C-reactive proteins. These results are displayed in Table 1. Red blood cell transfusion (two units) was performed with good hematological yield (Hg increase to 9.8 g/dL). The patient was admitted into the Internal Medicine ward for further etiologic study and stabilization.

**Table 1:** Blood test results at admission Severe hypochromic/microcytic anemia was detected, along with leucopenia, moderate hypoalbuminemia, and mildly elevated C-reactive protein.

Parameters	Results	Reference range
Hg (g/dL)	6.8	13.0-16.0
Hematocrit (%)	21.5	41-53
Mean corpuscular volume (fL)	71	83-103
Mean corpuscular hemoglobin (pg)	26.6	28-34
Leukocytes (x10^3^ /uL)	3.7	4.8-10.8
Neutrophils (x10^3^ /uL)	2.6	1.8-7.7
Eosinophils (x10^3^ /uL)	0.1	0.0-0.49
Basophils (x10^3^ /uL)	0.0	0.0-0.1
Lymphocytes (x10^3^ /uL)	0.7	1.0-4.8
Monocytes (x10^3^ /uL)	0.3	0.1-0.8
Albumin (g/dL)	2.4	3.4-5.0
C-reactive protein (mg/dL)	10.4	<5

While hospitalized, a serologic study comprising anti-HIV, HBsAg, anti-HCV, antinuclear antibodies, rheumatoid factor, and thyroid stimulating hormone was negative or within normal limits. Multiple stool microbiologic and parasitological studies were performed; however, all were negative. A thoracoabdominal computed tomography scan (CT) revealed bilateral pleural effusion, with a maximum thickness of four centimeters on the right side and two centimeters on the left. Multiple mesenteric, celiac, periportal, pancreatoduodenal, pericaval, iliac, and inguinal lymphadenopathies were visible, the largest being four centimeters in diameter.

**Figure 1:**
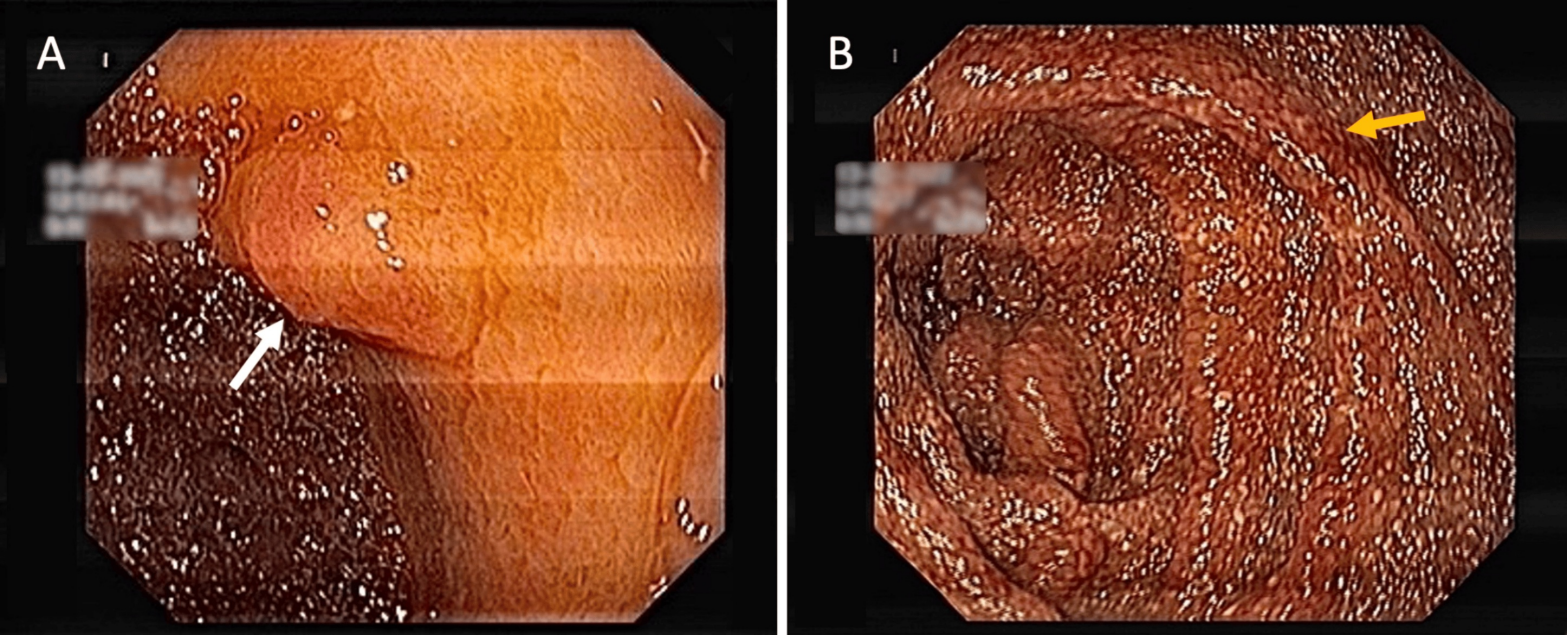
Upper endoscopic findings (A) Sessile lesion in the antrum (white arrow). (B) Duodenum with marked edema, congestion, and diffuse punctiform lymphangiectasia (yellow arrow).

**Figure 2:**
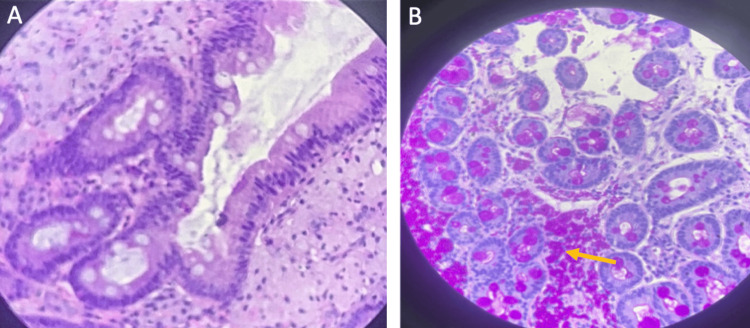
Histologic findings (A) Duodenum stained with hematoxylin-eosin, displaying macrophages in the lamina propria. (B) Periodic acid-Schiff (PAS)-stained macrophages (yellow arrow).

Diagnostic thoracocentesis was performed, revealing transudative effusion without malignant cells, as well as negative bacteriological pleural fluid analysis. Interferon-gamma release assay (IGRA) was also negative. Upper endoscopy was then performed, showing a sessile lesion measuring 12 mm in the antrum (Figure 1A). Progression of the endoscope into the duodenum revealed marked mucosal edema, congestion, and diffuse punctiform lymphangiectasia, and biopsy samples were obtained (Figure 1B). Histologic analysis unveiled spongy macrophages in the lamina propria using hematoxylin-eosin (HE) and PAS staining, suggestive of WD (Figures 2A, 2B).

For a definitive WD diagnosis, two inguinal lymph nodes were excised. Histologic analysis displayed overall preserved architecture, along with reactive alterations within the sinus, and interfollicular histiocytosis with a focus of epithelial cells in small nests. No neoplastic cells were present. Given these results, the diagnosis of WD was confirmed.

Following diagnosis confirmation, antibiotic therapy with intravenous ceftriaxone two grams/daily was initiated. After two weeks of intravenous therapy, the patient was discharged with an indication to complete antibiotic therapy with sulfamethoxazole/trimethoprim 800/160 milligrams every 12 hours for at least 1 year. Follow-up revaluation was scheduled in an outpatient Internal Medicine consultation.

Upon 12 months of antibiotic therapy, clinical improvement was remarkable as the patient fully recovered his previous weight. Fatigue, lower limb edema, and night sweats, as well as diarrhea, were no longer present. Blood tests showed complete resolution of hematological and biochemical abnormalities. Follow-up CT scan demonstrated a significant decrease in previously mentioned lymphadenopathy. Endoscopic follow-up was performed after one year of therapy, with duodenal biopsies revealing an absence of PAS-positive macrophages in histologic analysis.

## Discussion

WD is a rare disorder, with a higher prevalence reported in North America and Europe. The incidence rate is 1-3 cases per 1,000,000 people, with the mean age of symptom onset at 55 years. Males are more frequently affected, with a male/female ratio of 4-8/1 [3]. Transmission is usually through the fecal-oral route, as the bacillus has been identified in stagnant water, human feces, and soil [4]. WD susceptibility is thought to be associated with the HLA-B27 haplotype, similar to other infectious diseases, as only some carriers of the WD pathogen develop clinically overt disease. This suggests a probable complex interplay of bacterial, host, and environmental factors in its pathogenesis, with patients at risk of disease likely exhibiting some type of immune deficiency [5].

In WD, there is a massive infiltration of the intestinal mucosa with macrophages due to *T. whipplei* presence [5]. The bacteria is phagocytized by macrophages, which are unable to eliminate the internalized bacteria due to their ability to reduce the inflammatory response. Perpetuation of this process is accounted for by IL-16 secretion, increasing macrophage recruitment to sites of infection [2,5].

Due to the bacteria’s ability to infect different organs, clinical manifestations may be diverse thus making prompt diagnosis challenging.

Classic WD consists of three progressive stages: the first stage is characterized by musculoskeletal and constitutional symptoms such as arthralgia, arthritis, fever, and asthenia. The latter is followed by gastrointestinal, dermatological, and hematological signs and symptoms such as abdominal pain, diarrhea, weight loss, anemia, and edema. Ascites is an uncommon manifestation of WD, possibly resulting from malabsorption that leads to protein-losing enteropathy and hypoproteinemia, and a serum-ascites albumin gradient (SAAG) lower than 1.1. In the final stage, there is neurologic, ophthalmologic, and cardiac involvement [2,5]. A pathognomonic sign of neurological impairment due to WD is oculomasticatory myorhythmia, which consists of synchronous eye converging movements at the time of masticatory muscle contractions [6]. Since *T. whipplei *has a high affinity for lymph node tissue, lymphadenopathy is a common finding.

**Figure 3:**
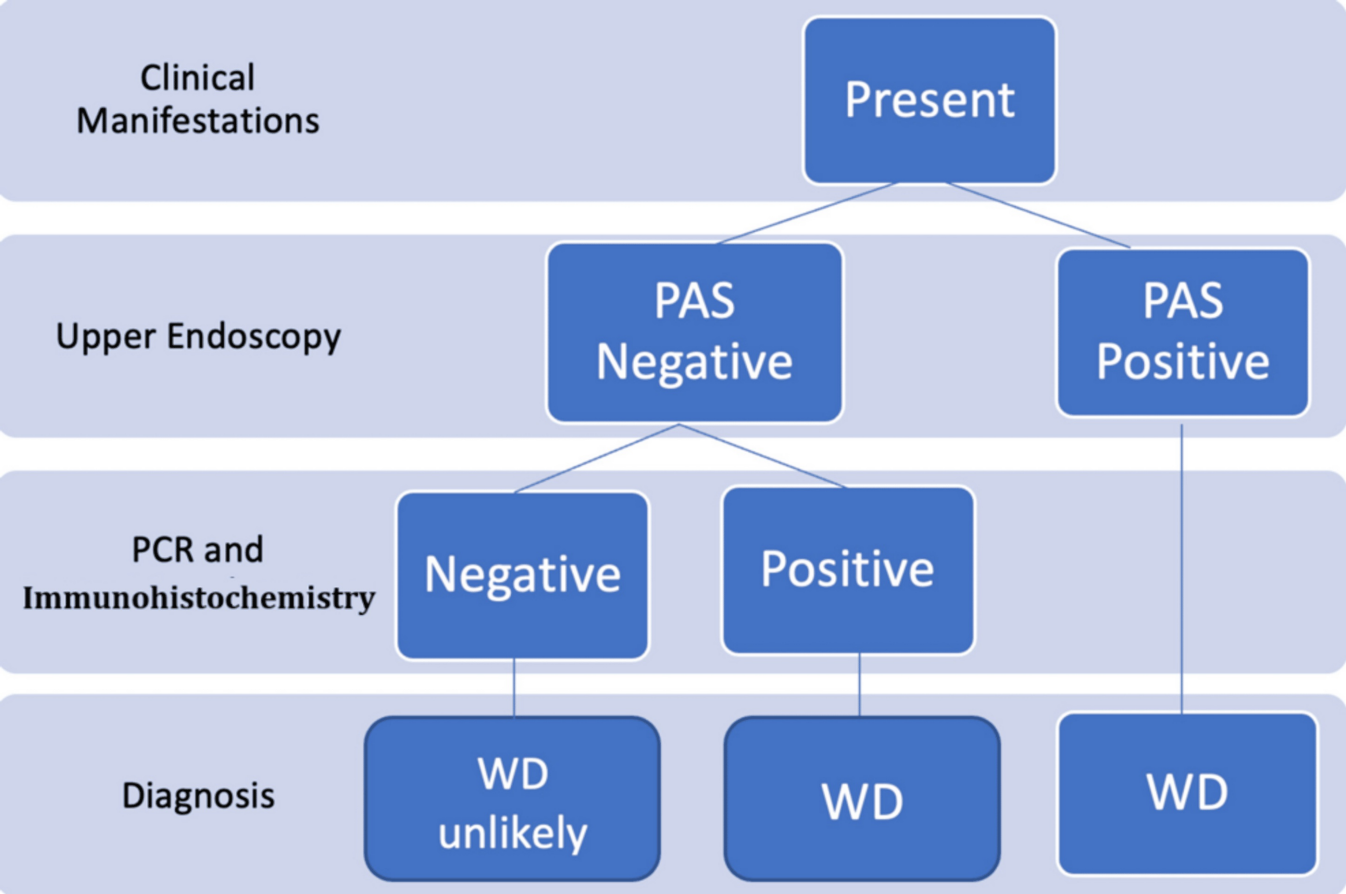
Diagnostic algorithm for Whipple's disease PAS: Periodic Acid-Schiff stain; WD: Whipple's disease

In upper endoscopy, macroscopic duodenal abnormalities include villi edema and widening, whitish plaques, and signs of duodenitis, mucosal erythema, or hemorrhage [7]. Definitive diagnosis typically requires histologic demonstration with PAS staining in macrophages from small bowel mucosa and/or polymerase chain reaction (PCR) identification of *T. whipplei* (Figure 3) [7].

Although the patient had been symptomatic for many months, this presentation of WD was considered of moderate severity, as no cardiac nor neurologic impairment was evident through clinical or imagiologic investigation. During diagnostic workup, many alternative diagnoses were excluded, specifically more frequent infectious causes (such as HIV, tuberculosis, hepatitis) as well as inflammatory/autoimmune etiologies. Furthermore, the presence of nonspecific imaging findings as well as transudative pleural effusion led to a diagnostic consideration of potentially rare causes. Macroscopic duodenal abnormalities in upper endoscopy raised clinical suspicion for WD diagnosis, and final confirmation was obtained with histologic analysis.

The patient's treatment plan is in accordance with current guidelines, which recommend a short course (two weeks) of either intravenous (IV) beta-lactam (ceftriaxone or penicillin (PCN) G) or meropenem (if PCN/cephalosporin allergy), followed by maintenance treatment with trimethoprim/sulfamethoxazole for one to two years, according to clinical response [1]. Follow-up with clinical, analytic, and endoscopic revaluation is recommended to ensure response to treatment and guide antibiotic duration.

## Conclusions

Whipple’s disease is a rare condition and its diagnosis is challenging. Nevertheless, as previously demonstrated, in patients with prolonged clinical presentations of gastrointestinal complaints associated with constitutional symptoms, clinicians should be aware of WD. Although in earlier stages, symptomatic presentation may be nonspecific, in advanced disease, some characteristic signs may prompt diagnosis. In particular, when common infectious/inflammatory etiologies are excluded, diagnostic workup should take into account rarer causes, further promoting WD exclusion. Upper endoscopy and subsequent histologic analysis with PAS staining or PCR detection of *T. whipplei *in duodenal samples are mandatory.

Thus, considering WD in the differential diagnosis of prolonged diarrhea is essential, as delays could lead to disease progression and multisystem involvement with possibly fatal outcomes.
